# Thermoregulatory responses in exercising rats: methodological aspects and relevance to human physiology

**DOI:** 10.1080/23328940.2015.1119615

**Published:** 2015-12-30

**Authors:** Samuel Penna Wanner, Thales Nicolau Prímola-Gomes, Washington Pires, Juliana Bohnen Guimarães, Alexandre Sérvulo Ribeiro Hudson, Ana Cançado Kunstetter, Cletiana Gonçalves Fonseca, Lucas Rios Drummond, William Coutinho Damasceno, Francisco Teixeira-Coelho

**Affiliations:** 1 Laboratório de Fisiologia do Exercício; Departamento de Educação Física; Universidade Federal de Minas Gerais; Belo Horizonte (MG), Brazil; 2 Laboratório de Biologia do Exercício; Departamento de Educação Física; Universidade Federal de Viçosa; Viçosa (MG), Brazil; 3 Laboratório de Fisiologia do Exercício; Universidade Estadual de Minas Gerais; Ibirité (MG), Brazil; 4 Centro de Formação de Professores; Universidade Federal do Recôncavo da Bahia; Amargosa (BA), Brazil

**Keywords:** body temperature, brain temperature, environment, exercise, heat, hyperthermia, physical exertion, rat, thermoregulation, treadmill running

## Abstract

Rats are used worldwide in experiments that aim to investigate the physiological responses induced by a physical exercise session. Changes in body temperature regulation, which may affect both the performance and the health of exercising rats, are evident among these physiological responses. Despite the universal use of rats in biomedical research involving exercise, investigators often overlook important methodological issues that hamper the accurate measurement of clear thermoregulatory responses. Moreover, much debate exists regarding whether the outcome of rat experiments can be extrapolated to human physiology, including thermal physiology. Herein, we described the impact of different exercise intensities, durations and protocols and environmental conditions on running-induced thermoregulatory changes. We focused on treadmill running because this type of exercise allows for precise control of the exercise intensity and the measurement of autonomic thermoeffectors associated with heat production and loss. Some methodological issues regarding rat experiments, such as the sites for body temperature measurements and the time of day at which experiments are performed, were also discussed. In addition, we analyzed the influence of a high body surface area-to-mass ratio and limited evaporative cooling on the exercise-induced thermoregulatory responses of running rats and then compared these responses in rats to those observed in humans. Collectively, the data presented in this review represent a reference source for investigators interested in studying exercise thermoregulation in rats. In addition, the present data indicate that the thermoregulatory responses of exercising rats can be extrapolated, with some important limitations, to human thermal physiology.

## Abbreviations

HLI: heat loss index
T_AMB_: ambient temperature
T_ABD_: abdominal temperature
T_BRAIN_: brain temperature
T_COL_: colonic temperature
T_CORE_: core body temperature
TNZ: thermoneutral zone
T_REC_: rectal temperature
VO_2_: rate of oxygen consumption
VO_2MAX_: maximum rate of oxygen consumption

## An Overview of the use of Rats for Studying Thermal Physiology During Physical Exercise

The study of physiological responses induced by physical exercise has received growing attention in the last decades, mainly due to the behavioral changes observed in modern human societies worldwide. Physical inactivity is a major risk factor for many chronic, non-communicable diseases and shortens life expectancy.^1^ The elimination of inactivity is estimated to increase the life expectancy of the Brazilian population by 0.31 years^2^ and the world's population by 0.68 y.^1^ Therefore, physical exercise, recently termed a real polypill,^3^ is currently accepted as a non-pharmacological strategy to treat these chronic, non-communicable diseases.

Environmental changes have also stimulated research on exercise-induced physiological responses, particularly thermoregulatory responses. Among the environmental changes that are currently being observed, global warming is considered one of the emerging concerns of the 21st century. The ambient temperature (T_AMB_; globally averaged) has elevated by 0.5°C since the mid-1970s, partially due to anthropogenic greenhouse gas emissions.^4^ Overall, global warming impacts the health and survival of several species.^5-7^ For example, epidemiological evidence suggests that progressive increases in T_AMB_ are directly related to increased hospitalizations due to cardiovascular disease and heat stroke.^8,9^ Thus, thermal physiology during exercise is an important topic of investigation, and the development of experimental models for studying this topic is welcome. In this context, a number of relevant experiments involving physical exercise and thermal physiology have been conducted using laboratory rodents.

The use of rodents in studies of thermal physiology is not a recent development. In the 1960s, Han and Brobeck studied thermoregulation in male rats bearing ventromedial hypothalamic lesions, an experimental model of hyperphagia.^10^ The colonic temperature (T_COL_) was measured as an index of the core body temperature (T_CORE_) as the rats were subjected to treadmill running; higher T_COL_ values after exercise were recorded in the lesioned rats than in the control rats.^10^ Despite the subsequent methodological advances, the use of laboratory rodents still represents an important experimental model in the field of exercise thermoregulation. The Resource Book for the Design of Animal Exercise Protocols published by the American Physiological Society in 2006 ^11^ claims that animals should be used whenever conducting an exercise physiology study in humans is inappropriate and whenever the animal research aims to improve the health of the animal itself. Examples of studies that might not be conducted in humans include lifetime investigations,^12,13^ studies that use invasive surgical procedures^14,15^ and studies using pharmacological tools to directly manipulate neurotransmission.^16-18^

The present review aims to provide insight and highlight recent advances in exercise thermoregulation. This review also intends to provide scientists with important methodological recommendations regarding thermoregulatory measurements in exercising rodents and to discuss the applicability of the outcomes yielded by these experiments with laboratory rodents to the understanding of human thermal physiology. Some of the relevant questions that will be addressed herein include the following: What is the most suitable experimental model for investigating exercise-induced thermoregulatory responses? What are the thermoregulatory responses in rodents that perform physical exercise? What are the main factors that interfere with these thermoregulatory responses? Can the thermoregulatory outcomes of these rodent experiments be extrapolated to human physiology? Most of the data discussed in the present review will be related to the thermoregulation of rats because although mice have been widely used in biological and medical research, few studies have investigated their thermoregulatory responses during physical exercise.

## Treadmill Running as the Experimental model for Investigating Thermal Physiology During Physical Exercise

The scientific literature describes different experimental models for studying exercise in rats, such as treadmill running,^19-21^ swimming,^22,23^ running wheels,^24,25^ weight lifting^26,27^ and climbing.^28,29^ This manuscript will focus on the thermoregulatory responses induced by treadmill running. The thermoregulatory responses induced by other exercise models, including swimming and wheel running, have been reported elsewhere^30,31^ but are beyond the scope of the present review.

Treadmill running allows for the precise and continuous measurement of effort intensity (i.e., oxygen consumption rate, heart rate or lactate concentration) and of thermoregulatory parameters (such as the T_CORE_ and skin temperatures). Moreover, the T_AMB_ can be easily adjusted to control environmental heat stress, and a large muscle mass is recruited during treadmill running; this recruitment can lead to high rates of heat production. Finally, other experimental models have important disadvantages that limit their feasibility for studying exercise thermoregulation. For example, it is not possible to measure the metabolic rate of swimming rats, and their tail skin temperature (T_TAIL_) will be greatly determined by the water temperature. Because water has higher specific heat and thermal conductivity than dry air, the thermoregulatory effects induced by exercise associated with water immersion may be huge.^32^ In fact, Harri and Kuusela showed that rats subjected to swimming training displayed some physiological adaptations that were similar to those induced by cold exposure.^33^ For exercise on running wheels, animals can often decide when to start or finish running and at which speed to run. Therefore, the exercise performed in running wheels represents a voluntary type of exercise, and the effort intensity is self-paced. Similar to the situation in swimming rats, it can be challenging to obtain precise and continuous measurements of the metabolic rate and cutaneous heat loss in rats that are voluntarily running on a wheel. Lastly, resistance exercise protocols augment the metabolic rate, but usually for short periods; therefore, such protocols do not provoke major changes in the T_CORE_ and thermoeffector activity.

## Typical Thermoregulatory Responses of Rats Subjected to Treadmill Running

**Figure 1.**
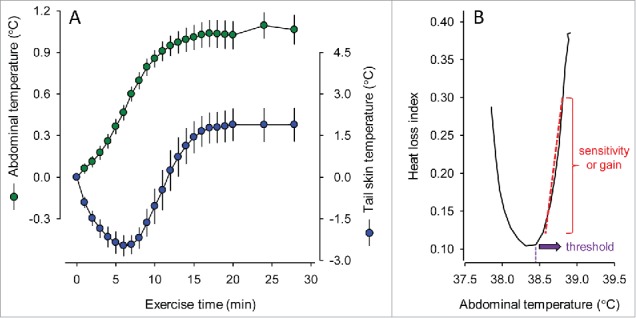
Abdominal and tail-skin temperatures of rats subjected to a 45-min period of treadmill running at a constant speed of 18 m/min and an ambient temperature of ∼24°C. These experiments were conducted under conditions of compensable heat stress (moderate-intensity exercise in a temperate environment). The data used to plot this graph were taken from experiments reported in the following manuscript: Wanner SP, Leite LH, Guimarães JB, Coimbra CC. Increased brain L-arginine availability facilitates cutaneous heat loss induced by running exercise. Clin Exp Pharmacol Physiol 2015; 42:609-16. Copyright © 2015 John Wiley and Sons. Used with permission.

During constant-speed, moderate-intensity treadmill running performed at a moderate T_AMB_ (i.e., range of 21 to 24°C), the thermoregulatory response of an exercising rat can be didactically divided into 2 distinct phases, namely, the dynamic and steady-state phases^34,35^ (**Fig. 1A**). Each phase has distinct patterns of heat production and heat loss; thus, the resulting changes in T_CORE_ are quite different between these 2 phases.

At the beginning of an exercise session, the heat production abruptly increases, which results from an augmented metabolic rate in the working muscles.^35^ This higher metabolic heat production is not immediately counteracted by a higher rate of heat loss. Additionally, the T_TAIL_ decreases during the first minutes of exercise, suggesting the occurrence of skin vasoconstriction.^34,36^^,^^37^ Consequently, the T_CORE_ rapidly and exponentially increases in response to the initiation of exercise, with the rates of T_CORE_ increase attaining values of approximately 0.11°C/min.^38^ Controversy remains regarding the mechanism underlying the initial skin vasoconstriction. The most accepted hypothesis claims that blood flow is transiently deviated from skin vessels (and from visceral and renal beds) to the muscular beds, with the degree of this cutaneous vasoconstriction closely related to the exercise intensity and active muscle mass.^39^ However, limited evidence rules out the possibility that the exercise-induced increase in the T_CORE_ is a consequence of an increase in the set point at which T_CORE_ is regulated^40^ rather than a consequence of the blood flow redistribution.

After the initial moments of exercise (∼10 min), T_TAIL_ begins to markedly increase until it reaches a plateau that is sustained until physical exertion is interrupted.^34,36^^,^^37^ This increase in T_TAIL_ indicates that cutaneous vasodilation mechanisms are activated and leads to the second phase of thermal balance during exercise, namely, the steady-state phase. In this phase, the rates of heat production and heat loss will stabilize at similar levels, and the T_CORE_ will therefore reach a plateau. Thereafter, the T_CORE_ will be stably maintained at a high level or will slightly increase until exercise cessation.

A steady-state T_CORE_ is usually observed during exercise under compensable heat stress conditions (e.g., running at a speed of 18 m/min at an ambient temperature of 21°C). In fact, the T_CORE_ value attained during the steady-state phase is dependent on the threshold T_CORE_ for cutaneous heat loss.^36^ Nevertheless, this profile is not a universal response induced by physical exercise, and the T_CORE_ will not reach a plateau under non-compensable heat stress conditions, particularly when rats are subjected to very intense physical efforts or to physical efforts at high temperatures.

Similar kinetics are observed for the exercise-induced thermoregulatory responses in humans under compensable heat stress conditions. At the onset of dynamic exercise, the rate of metabolic heat production is rapidly elevated, with a time constant of ∼5 min.^41^ The increased heat production at exercise onset is not immediately matched by increased whole-body heat loss.^42^ Therefore, the mean body temperature increases to levels higher than the onset threshold values, thereby activating autonomic heat loss responses characterized by cutaneous vasodilatation and sweating.^43^ The elevation in whole body heat loss caused by the activation of both autonomic thermoeffectors has a time constant of ∼10 min.^41,44^ Interestingly, when heat balance was evaluated in human skeletal muscles (not at the whole body level) at the onset of intense dynamic exercise, similar findings were observed. The rate of heat production increased linearly during a 2-min leg extension exercise, whereas the rate of muscular heat removal by the blood was not significant until after 10 s of exercise, when convective heat removal started to increase progressively.^45^ Therefore, the rate of heat storage in the knee extensors was greatest early in the exercise and then gradually declined.^45^

Regarding rat thermoregulation, tail-skin vasodilation is the primary heat loss mechanism in this species,^46-48^ although vasodilation of the skin of the feet,^49,50^ the evaporation of saliva spread onto the body surface,^51,52^ the evaporation of water from the respiratory tract,^53^ and even voluntary urination associated with urine spreading activity^54^ may also contribute to the total heat dissipation. A study employing calorimetry indicated that the rat tail receives a considerable amount of heat corresponding to 40% of the basal metabolic rate.^48^ The tail skin blood flow is modulated by noradrenergic vasoconstrictor activity,^47,55^ and there is evidence that trunk sympathectomy elevates the tail blood flow to the levels observed in hyperthermic rats.^47^ Therefore, it is reasonable to propose that exercise-induced skin vasodilation in rats results from tail sympathetic activity withdrawal.

Saliva-spreading behavior is an important adjunct thermolytic mechanism for T_CORE_ regulation, particularly in hot environments with a decreased thermal gradient between the skin and ambient.^56^ However, the importance of this thermolytic pathway is minor during treadmill running because the animals are unable to spread saliva over their fur to facilitate evaporative cooling.^37,57^ Therefore, the most effective way for a rat to regulate its T_CORE_ while running at different work levels or at high ambient temperatures is to increase the blood flow in its tail skin.^57,58^ Other means for dissipating stored heat, such as the evaporation of water from the respiratory tract, may also contribute to thermoregulation in a running rat. Tanaka et al.^53^ showed that evaporative water loss increases in proportion to exercise intensity. Nevertheless, the relative contribution of evaporation to the global heat loss of a running rat has not been determined.

A common approach to analyze thermoeffector activity is to graph its response (e.g., skin blood flow) as a function of the T_CORE_. In this way, a threshold T_CORE_, i.e., the temperature at which the onset of the thermoeffector response occurs, can be identified, and the slope of the post-threshold relationship can be analyzed to obtain an index of the sensitivity/gain of the thermoeffector to further increases in the T_CORE_ (**Fig. 1B**). In these analyses, it is important to recognize that the skin surface temperature can also influence the responsiveness to increases in the T_CORE_; high skin temperatures augment responsiveness, and low skin temperatures have the opposite effect.^59^ In fact, in experiments designed for studying thermal physiology in humans, the thermoeffector response is commonly plotted against the mean body temperature,^60,61^ which is calculated using T_CORE_ and skin surface temperature values.

Because of the problems with comparing skin sympathetic nerve activity between individuals or over separate days,^62^ the onset threshold and sensitivity of thermoeffector responses currently represent the most viable means for evaluating the physiology of temperature regulation.^63^ Changes in both the threshold and the sensitivity can represent a central and/or peripheral modulation of temperature regulation; however, changes in the onset threshold are traditionally considered indicators of central modulation,^60^ whereas changes in sensitivity describe peripheral adaptations in thermoeffector responses.^64,65^

In agreement with the model proposed above, previous studies showed that the pharmacological manipulation of different neurotransmitters by directly injecting small amounts of a drug into the brain changes the threshold for cutaneous heat loss without inducing corresponding changes in sensitivity. Several pharmacological manipulations, such as the blockade of nitric oxide synthase,^34^ AT1 receptors for angiotensin^17^ and muscarinic cholinoceptors,^66^ increase the heat loss threshold. In contrast, the increased availability of brain L-arginine, the precursor for NO synthesis, decreases the heat loss threshold.^36^ Importantly, all of these drugs were injected in small volumes (2 µl or 0.2 µl when the drugs were injected into the cerebral ventricles or into a nucleus, respectively) and likely remained restrained within the central nervous system, not reaching the body periphery.

## Factors that Influence Running-induced Changes in Thermoregulation

"Although the rat has been used extensively in exercise studies, regulation of body temperature during exercise in this species remains a puzzling topic for work physiologists." The latter sentence was written by Tanaka et al.^53^ in 1988 and remains valid almost 30 y later, particularly regarding the relationship between exercise intensity and T_CORE_. This section will discuss the influence of exercise intensity and some other factors on changes in the thermoregulation of exercising rodents and will provide an updated description of exercise thermoregulation in rats based on recently published results.

### Site / method of temperature measurement

The most used theoretical model to explain human thermoregulation consists of a 2-compartment model, which estimates an average body temperature by separating the body temperature into a "core" compartment temperature (which comprises the abdominal, thoracic and cranial cavity temperatures) and a "shell" compartment temperature (which comprises the skin, subcutaneous tissue and muscle temperatures).^67^ Therefore, the mean body temperature of humans is calculated using an equation including weighted T_CORE_ and T_SKIN_ values, with the weight attributed to each temperature index being dependent on the environmental conditions.^67^ However, the use of a 2-compartment model is not universally accepted; for example, Jay et al.^68^ proposed the use of a 3-compartment thermometry model that includes the thermal influences of muscles and that appears to yield a more precise estimate of the average body temperature compared with the estimate using a 2-compartment model.

In the case of rats, the average body temperature is not commonly calculated; experiments using this species usually analyze the T_CORE_ and skin temperatures separately. The T_CORE_ (or deep body temperature) is measured in the inner tissues of the body, the temperatures of which are not changed by circulatory adjustments that allow heat dissipation to the environment and that affect the thermal shell of the body. ^69^ The brain is generally accepted as a section of the thermal core^69^ even though the brain temperature (T_BRAIN_) in several mammalian species may deviate to some extent from the temperature of the remaining thermal core region due to selective brain cooling.^70,71^ In contrast, the shell temperature corresponds to the skin and subcutaneous tissue temperature (and possibly the muscle temperature). The skin and mucosal surface temperature may deviate from the T_CORE_ due to heat exchange with the environment and to changes in the circulatory convection of heat from the core to heat-exchange surfaces.^69^

Although the temperatures of the inner body tissues are not directly changed by circulatory adjustments that allow heat loss to the environment, these temperatures are not homogeneous and do not respond in a similar manner (particularly regarding their time course) to several arousing stimuli.^72^ Baseline T_CORE_ values measured using rectal / colonic probes are approximately 0.85 to 1.20°C higher than the abdominal temperature (T_ABD_) values measured by telemetry.^73^ Despite this difference in baseline values, both the telemetry and rectal / colonic probe methods are able to reflect the hyperthermic or hypothermic effects induced by different drugs, and high positive correlations were observed between the 2 methods, suggesting that either method could be used in most studies of temperature regulation.^73^

The exercise-induced changes in the T_COL_ [or rectal temperature (T_REC_)] and the T_ABD_ have never been simultaneously recorded in an exercising rat. The relationship between these 2 temperatures is relevant to a better understanding of the outcomes of different studies because the first exercise thermoregulation experiments, which were performed in the 1960s, 1970s and 1980s, used the T_COL_ as an index of the T_CORE_. The use of telemetry for measuring the T_ABD_ as an index of the T_CORE_, which started in the 1990s, is certainly more appropriate in studies consisting of long-term measurements, for which the animals must remain undisturbed.^74^

**Figure 2.**
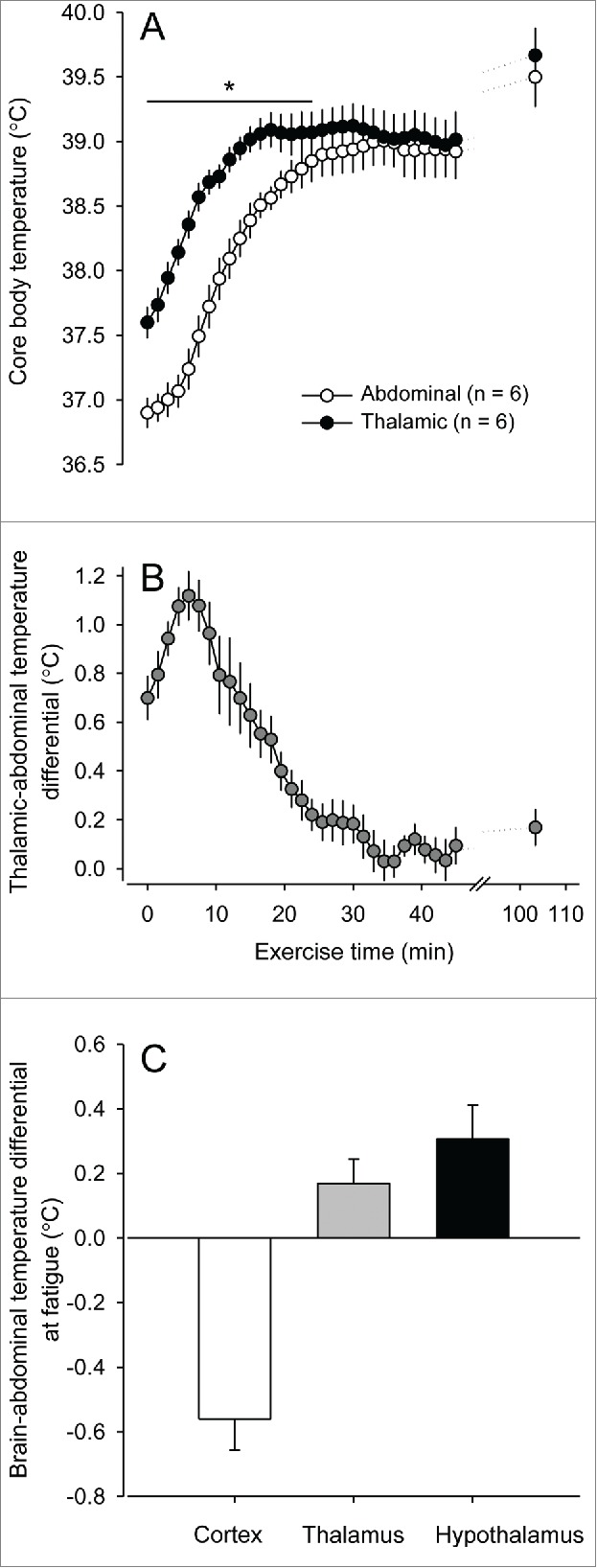
Thalamic and abdominal temperatures (**A**) and the thalamic-abdominal temperature differential of rats (**B**) subjected to constant-speed treadmill running until fatigue. * denotes a significant difference (p< 0.05) compared with the abdominal temperature. Panel **C** shows the brain-abdominal temperature differential of running rats at volitional fatigue. The brain temperature was measured at different sites (i.e., the brain cortex, thalamus and hypothalamus). All of these experiments were conducted in temperate environments (ambient temperatures ranging from 24 to 25°C). Please note that these temperature differentials were obtained from experiments with distinct exercise intensities and protocols. The data measured were taken from the experiments reported in the following manuscripts: (i) thalamus - Damasceno WC, Pires W, Lima MR, Lima NR, Wanner SP. The dynamics of physical exercise-induced increases in thalamic and abdominal temperatures are modified by central cholinergic stimulation. Neurosci Lett 2015; 590:193-8. © 2015 John Wiley and Sons. Reproduced by permission of John Wiley and Sons. Permission to reuse must be obtained from the rightsholder; (ii) hypothalamus - Fonseca CG, Pires W, Lima MR, Guimarães JB, Lima NR, Wanner SP. Hypothalamic temperature of rats subjected to treadmill running in a cold environment. PLoS One 2014; 9:e111501. Open-access manuscript; (iii) brain cortex - Drummond LR *et al.*, *unpublished data*. The rats were subjected to an incremental-speed exercise at an ambient temperature of 25°C. The brain cortex temperature was measured using a thermistor inserted at the following stereotaxic coordinates: anteroposterior: 3 mm anterior to the bregma; mediolateral: 3 mm right to the midline; and dorsoventral: 1.8 mm dorsal to the skull. The abdominal temperature was measured by telemetry. This experiment was approved by the Ethics Commission for Animal Use of the Universidade Federal de Viçosa (Brazil; protocol number 58/2012).

We recently compared the kinetics of exercise-induced increases in the T_BRAIN_ and T_ABD_ in rats subjected to treadmill running. At the beginning of constant-speed running at 23-25°C, the T_BRAIN_ increased more rapidly than did the T_ABD_. This observation was true irrespective of whether the T_BRAIN_ was measured in the cortex (Drummond LR et al., *unpublished data*), thalamus^75^ (**Fig. 2A**) or hypothalamus.^76^ This early difference in the rate of increase of the T_CORE_ indexes likely resulted from intra-brain heat production rather than from the delivery of warm peripheral blood, as indicated by the increase in the brain-abdominal temperature differential. In addition, exercise promotes increased sympathetic outflow to the visceral vascular beds, which induces vasoconstriction and therefore allows a blood flow redistribution that is essential for the organism's ability to meet the increased demands of the active muscles, lungs and brain for oxygen and energetic substrates.^77^ Underperfusion of the abdominal viscera (which is not highly active during exercise) may limit the delivery of the heat produced by the active organs and tissues, thus slowing the rate of increase in the T_ABD_ and contributing to an increase in the brain-abdomen temperature differential at the beginning of exercise.

The temperature inside the brain is not homogenous, and a dorsoventral gradient has been reported in resting rats, with higher temperature values observed in ventral areas than in dorsal areas of the brain.^38,78^^,^^79^ During exercise, the hypothalamic temperature was higher than the T_ABD_ throughout the exercise period, including a 0.3°C-higher value at volitional fatigue.^76^ Similarly, the thalamic temperature was never lower than the T_ABD_ during a fatiguing exercise (**Fig. 2A**). At the beginning of the exercise, the thalamic temperature was 0.7°C higher than the T_ABD_, and the largest difference between these T_CORE_ indexes was 1.1°C, which was recorded as early as the 6th min of exercise. Subsequently, this thalamic-abdomen temperature differential decreased, approaching 0 after 30 min of treadmill running^75^ (**Fig. 2B**). Interestingly, the T_BRAIN_ measured in the brain cortex was lower than the T_ABD_ at the final moments of exercise and at volitional fatigue (Drummond LR et al., *unpublished data*), which is a quite different response compared with the measurements made in the thalamus or hypothalamus (**Fig. 2C**). Hasegawa et al. previously demonstrated that the brain cortex temperature was lower than the T_ABD_ at volitional fatigue.^16^ Taken together, these findings suggest that the T_BRAIN_ value and the brain-abdomen temperature gradient are dependent on the site/depth of T_BRAIN_ measurement. Moreover, the selective brain cooling phenomenon might occur in specific brain areas rather than the whole brain. Further studies with measurements of the temperature at multiple brain sites in the same exercising rat are warranted.

In our recent studies,^75,76^ the T_ABD_ and T_BRAIN_ were simultaneously measured using telemetric devices and thermistors, respectively. This fact raises an important methodological issue because telemetric devices have high thermal inertia and may not accurately measure rapid changes in the T_ABD_.^80^ Therefore, the methodology employed here may have overestimated the faster increase in the T_BRAIN_ that occurred in response to exercise initiation. However, our observations support the findings from studies that measured the abdominal aorta and brain temperatures with thermocouples in rats subjected to several arousing stimuli; these rats exhibited faster increases in the T_BRAIN_ than in the temperature of the abdominal aorta.^78^

Another relevant question regards the site used for measuring the skin temperature. The tail surface is the most common site for measuring the skin temperature, and many investigators associate this temperature with tail-skin blood flow.^47,81^ The evaporative heat loss from tail skin is negligible, and, therefore, water evaporation does not influence the T_TAIL_ value in a running rat. Thus, the T_TAIL_ measurement is a valid measurement for determining the dry heat loss from the tail skin to the environment. This measurement becomes even more precise when the heat loss index (HLI) is calculated to minimize the effects of the T_AMB_ or T_CORE_ on changes in the tail-skin temperature. The heat loss index consists of a ratio between 2 thermal gradients and is calculated by dividing the tail skin-ambient temperature differential by the core-ambient temperature differential. This index varies from 0 (when the T_TAIL_ is equal to the T_AMB_, indicating cutaneous vasoconstriction) to 1 (when the T_TAIL_ is equal to the T_CORE_, indicating vasodilation).^82,83^ However, this upper limit is theoretical, and HLI values above 0.7 have not been recorded in a running rat.

Thermocouples are often placed on the lateral surface of the tail to measure the T_SKIN_.^15,66^^,^^84,85^ This position was selected based on observations of the considerable amount of blood flow in the 2 major lateral veins when the tail is heated.^48^ However, measurement of the T_SKIN_ using thermocouples attached to the dorsal surface of the tail is also typical^17,34^^,^^36,53^ and provides T_SKIN_ results similar to those of experiments measuring the temperature at the lateral surface of the tail. In fact, a more recent study histologically re-examined rat tail vascularization and showed the presence of subcutaneous veins in the dorsal aspect of the tail.^86^ The number and size of these veins are not uniform along the entire length of the tail; in the proximal segment, where the T_TAIL_ is usually measured, a small vein is present on each side close to the tail midline.^86^

### Influence of exercise intensity, duration and protocol

The exercise intensity, duration and protocol are among the factors that interfere with the exercise-induced changes in body temperature. In experiments performed in the treadmill setup, the exercise intensity is dependent on the treadmill speed or incline, whereas the exercise duration is determined by the running time, and the exercise protocol refers to the possible combinations of intensity and duration (e.g., constant-speed exercise, incremental-speed exercise and intermittent exercise).

The finding that the magnitude of hyperthermia is dependent on the exercise intensity seems obvious. To guarantee an adequate energy supply for active muscles during physical exertion, the body metabolism is accelerated based on the absolute exercise intensity. The absolute exercise intensity refers to an exercise intensity that requires a given amount of energy expenditure, expressed as kcal/min or mLO_2_/min (e.g., a rat running at a constant speed of 15 m/min). As a byproduct of the augmented metabolic rate, heat is generated in the contracting muscles and then dissipated to the other body tissues by conduction or through circulating blood. In humans, the T_CORE_ increases in proportion to the work intensity over a wide range of T_AMB_ values.^87^

In running rodents, the magnitude of exercise-induced increases in the T_CORE_ is also positively associated with the exercise intensity. This positive association indicates that a higher exercise intensity is related to a higher increase in the T_CORE_. However, this is a not a universal finding; some researchers^57^ have failed to reliably demonstrate the proportional relationship between the steady-state T_CORE_ and workload that is so clearly found in humans.^87-89^ In fact, intensity-dependent hyperthermia is clearly observed during a single exercise bout consisting of multi-stage, incremental-speed treadmill running.^90,91^ However, the effects of the exercise duration and of accumulated physical exertion, particularly at higher speeds, on the magnitude of hyperthermia cannot be ruled out in this exercise protocol.

Gollnick and Ianuzzo^90^ performed one of the first experiments that measured the T_CORE_ in running rats. These researchers showed that the T_COL_ values detected during exercise were higher than the pre-exercise values and that the T_COL_ increased as a function of the exercise intensity during incremental-speed treadmill running. Moreover, an individual recording presented in their manuscript clearly showed that the T_COL_ plateaued while the rat ran at 1.0 mph and 1.33 mph. In contrast, a sharp increase in temperature was observed at a running speed of 2.0 mph, with the animals becoming fatigued within a short period of time.^90^

The above-mentioned findings were reproduced in subsequent studies. Shellock and Rubin^57^ observed increases in both the T_COL_ and rate of oxygen consumption (VO_2_) during successive incremental exercise work rates at cooler ambient temperatures, with a highly linear relationship between the T_COL_ and VO_2_ (pooled correlation coefficient of 0.91). More recently, Hasegawa et al.^91^ subjected rats to 1 h of incremental-speed treadmill running at 23°C. The treadmill speed was increased every 20 min (10, 20 and 26 m/min). Relative to the resting T_CORE_, the T_CORE_ immediately increased with exercise at the lowest exercise intensity (10 m/min). In addition, treadmill running increased the T_CORE_ in a linear manner at the second (20 m/min) and final (26 m/min) stages of exercise such that the T_CORE_ at the end of each exercise stage was significantly increased from that at the previous stage. Similar findings were observed with the VO_2_ measurements.^91^

**Figure 3.**
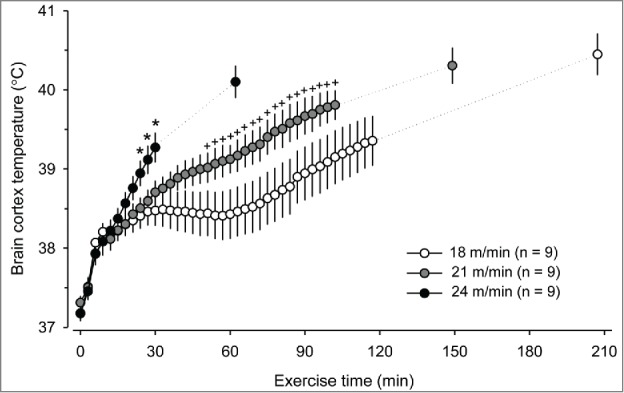
Cortical brain temperature of rats that were subjected to treadmill running until fatigue at 3 different speeds (18, 21, and 24 m/min). The ambient temperature was controlled at 25.2 ± 0.2°C by the use of air conditioning. * denotes a significant difference (p< 0.05) compared with the 21 m/min and 18 m/min trials. + denotes a significant difference (p < 0.05) compared with the 18 m/min trial. The data used to plot this graph were taken from the control experiments reported in the following manuscript: Kunstetter AC, Wanner SP, Madeira LG, Wilke CF, Rodrigues LO, Lima NR. Association between the increase in brain temperature and physical performance at different exercise intensities and protocols in a temperate environment. Braz J Med Biol Res 2014; 47:679-88. Open-access manuscript.

However, when rats were subjected to a single-stage exercise in the study of Shellock and Rubin,^57^ no statistically significant increases in the T_COL_ related to the progressively higher treadmill speeds or levels of VO_2_ were observed. At the 16th minute of exercise, the rats subjected to running at a constant speed of 18 m/min and no incline had a T_COL_ similar to that of rats running at 25 m/min and 5% incline.^57^ Therefore, the researchers concluded that rats do not reliably exhibit the proportional relationship between the steady-state T_CORE_ and work. However,^57^ some research groups have identified an association between hyperthermia and exercise intensity during single-stage treadmill running.^37,38^^,^^92^ For example, a trend toward higher T_COL_ with increasing speed was evident in the study conducted by Wilson et al.^37^ In addition, the increase in T_BRAIN_ clearly differed among the 3 running speeds (18, 21 and 24 m/min), as evaluated by Kunstetter et al.^38^ (**Fig. 3**). At the speed of 18 m/min, equilibrium was attained between the rates of heat production and heat loss, and a plateau in the T_BRAIN_ was therefore observed. In contrast, a temperature plateau was never observed at 21 and 24 m/min.^38^ These high intensities likely provoked elevated rates of heat production that overcame the ability of rats to dissipate heat, causing the T_BRAIN_ to constantly increase during exercise.

The initial increase observed in the T_CORE_ of rats in response to physical exercise appears to not be specific to the exercise protocol and intensity (**Fig. 3**). When measuring the T_BRAIN_ of rats subjected to 3 different running speeds, we observed similar rates of T_BRAIN_ increases during the first 8 minutes of exercise.^38^ During the initial phase of exercise, i.e., up to the 8^th^ minute, the rats exhibited the highest rates of T_BRAIN_ increases, which were independent of the treadmill speed. This marked T_BRAIN_ increase was likely caused by animal handling, a stressful procedure that includes inserting the brain thermistor prior to physical exertion.

Because the magnitude of hyperthermia in rats depends on the exercise intensity, as demonstrated by most studies,^37,38^^,^^92^ the next important issue to address is whether this hyperthermia magnitude is determined by the absolute or relative exercise intensity. The explanation for the absolute exercise intensity was provided earlier. The relative exercise intensity refers to an exercise intensity relativized by the maximum rate of oxygen consumption (%VO_2MAX_) or the maximum treadmill speed attained by an individual or animal.

In a seminal study performed with humans, Saltin and Hermansen^93^ described that at a given room temperature, the changes in the T_CORE_ are related to the relative exercise intensity, which is expressed as a percentage of the VO_2MAX_. The findings of Saltin and Hermansen^93^ were re-examined almost 50 y later. Gant et al.^94^ confirmed that the magnitude of hyperthermia in humans subjected to treadmill running was associated with the relative rather than the absolute exercise intensity. When comparing the physiological responses of 2 groups (i.e., one group with moderate VO_2MAX_ and another with higher VO_2MAX_) during a 60-min period of running at 60% of the VO_2MAX_, no differences were observed in the exercise-induced increases in the T_CORE_ and heart rate.^94^ However, when these 2 groups were subjected to the same running speed (similar absolute exercise intensity), the group with moderate VO_2MAX_ exhibited higher increases in the T_CORE_ and heart rate than did the group with high VO_2MAX_.^94^

Tanaka et al.^53^ performed a very well-designed study to characterize the thermoregulatory responses of running rats relative to the work intensity, which was expressed as a percentage of the VO_2MAX_. At a T_AMB_ of 24°C, the T_REC_ at the beginning of tail-skin vasodilation increased in proportion to the exercise intensity at a range from 45% to 75% of the VO_2MAX_; at intensities higher than 75% of the VO_2MAX_, this linear, positive association levelled off. After tail vasodilation, the T_REC_ remained steady and was also proportional to the work intensity (in the same range as described above) at the end of the 30-min period of exercise. Thus, the T_CORE_ of running rats increases in proportion to the relative exercise intensity.

Exercise thermoregulation is also influenced by the duration of treadmill running. The changes in the T_CORE_, heat production and heat loss that occur during the initial minutes of exercise have been previously discussed in detail; therefore, we will now highlight the effects of the duration of exercises lasting more than 30 min. Even when performed under conditions of compensable heat stress, the T_CORE_ begins to increase again after a plateau is reached. For example, Kunstetter et al.^38^ reported that rats subjected to a constant running speed of 18 m/min exhibited a plateau in the T_BRAIN_ that lasted approximately 40 min and was followed by a second, clear increase in the T_BRAIN_. This second increase in the T_CORE_ may result from either a gradual reduction in the running economy (i.e., the ratio of the power output to VO_2_), which causes a subsequent increase in the heat production rate, or to augmented exposure to electrical stimulation, which causes a subsequent increase in the sympathetic outflow that promotes heat conservation and thermogenesis.

Another important factor that influences exercise thermoregulation is the protocol employed during treadmill running. In a comparison of the thermal responses between incremental and constant-speed running, the T_BRAIN_ from the 24th minute to the end of the incremental running protocol was lower than the T_BRAIN_ during constant exercise at 24 m/min.^38^ The influence of the running protocol on the T_BRAIN_ increase may result from differences in the evolution of exercise intensity, which is an inherent characteristic of each running protocol. During the initial stages of the incremental exercise protocol, the power output by the animals and, consequently, the rate of heat production were low (e.g., it took 42 min for rats to begin running at 24 m/min). However, even when the animals achieved high speeds during the final stages of the incremental exercise protocol, their T_BRAIN_ values remained lower.

Collectively, the data indicate the influence of exercise intensity, duration and protocol on the changes in the body temperature of rats subjected to treadmill running. Therefore, a researcher should be aware of the outcomes of his/her choices before selecting the most suitable exercise. If a marked increase in T_CORE_ is desirable to investigate the association between thermoregulation and physical performance, incremental-speed treadmill running is not the most suitable experimental protocol.

### Influence of the environmental conditions

Despite the importance of the 3 factors mentioned in the previous section, environmental conditions, particularly the T_AMB_, are the most important modulators of changes in the body temperatures of exercising rats.

The dry ambient temperature is one of the parameters that is evaluated to determine the environmental conditions. Aside from temperature, the relative humidity, wind speed and radiation represent alternative parameters that modulate the heat exchange between a body and the ambient and thus contribute to the overall environmental thermal stress. In experiments with resting rats, the environmental conditions are usually classified as thermoneutral, subneutral or supraneutral.^82^ According to the more recent glossary of terms for thermal physiology, the term thermoneutral zone (TNZ) refers to the range of ambient temperatures at which temperature regulation is achieved only by the control of sensible heat loss, without regulatory changes in metabolic heat production or evaporative heat loss.^69^ In this context, the use of terms such as cool, cold, moderate, warm and hot is not adequate for describing an environmental condition; these terms are suitable for describing a thermal sensation.

Several research groups have attempted to identify the range of ambient temperatures associated with the TNZ. Nevertheless, because a neutral T_AMB_ measured in a given experimental setup cannot be used as a standard for experiments conducted in other experimental setups,^82^ the results reported by different laboratories are contradictory. Several research groups^95,96^ determined the rat TNZ to be between 28 and 34°C, but other groups reported lower temperature ranges from 22 to 27°C^97^ or from 18 to 28°C.^98^ Accounting for the more recent concept of the TNZ,^69^ Romanovsky et al.^82^ suggested the use of skin thermometry, which is a definition-based, simple, and inexpensive technique, to determine whether the experimental conditions are neutral, subneutral, or supraneutral for a given animal. Using skin thermometry and under the conditions tested (rats placed in confiners, without bedding or filter tops and without the possibility of group thermoregulation), the ambient temperature range of 29.5 to 30.5°C satisfied the TNZ criteria in Wistar rats.^82^

Using the method proposed by Romanovsky et al.,^82^ we observed HLI values ranging from 0.20 to 0.30 in a resting rat maintained inside the chamber that contained the treadmill belt (with the electrical fan off) at a local T_AMB_ of 24-25°C.^36^ Similar findings (average HLI values of 0.23) were reported by Lima et al.,^99^ who maintained the rats inside the same chamber, but with the electrical fan on and at a local temperature of 26°C.^36^ Collectively, these data suggest that ambient temperatures ranging from 24 to 26°C correspond to the lower extremity of the TNZ of rats in the treadmill setup.

The concept of thermoneutrality does not apply to ectotherms (i.e., bradymetabolic species) and is not suitable for exercising endotherms because the increased metabolic rate caused by muscular contractions is an inherent characteristic of physical exercise. It would be counterintuitive to state that a rat runs under thermoneutral conditions while displaying a T_CORE_ value of 39°C and a metabolic rate that is five-fold higher than the resting metabolic rate. Considering the latter statement, authors usually do not describe the environment as thermoneutral during exercise experiments and use alternative descriptions, including temperate environments^38^ or normal T_AMB_.^100,101^

The increase in the T_CORE_ of exercising humans is largely dependent on the power output but is largely independent of a wide range of ambient temperatures.^87,88^^,^^102^ The term prescriptive zone is used to describe the range of ambient temperatures at which humans can achieve a steady-state response for the T_CORE_, with the level of steady-state T_CORE_ dependent on the metabolic rate (either absolute or relative exercise intensity) and independent of the environment within a range of compensable heat stress conditions. Of note, the linear relationship between the metabolic rate and the T_CORE_ is valid for a given person (controlling for factors such as heat acclimation and hydration status) but does not always hold for comparisons between different people.

**Figure 4.**
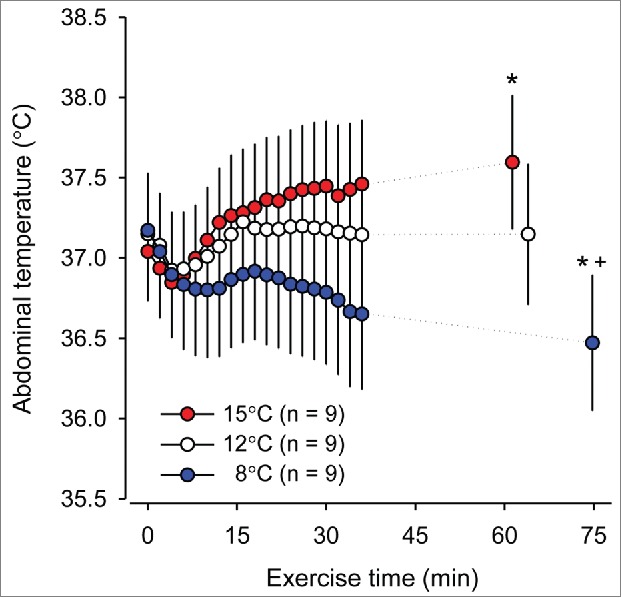
Abdominal temperature of rats subjected to treadmill running until fatigue at a constant speed of 20 m/min at 3 ambient temperatures (i.e., 8, 12 and 15°C). * denotes a significant difference (p< 0.05) compared with the 12°C trial. + denotes a significant difference (p < 0.05) compared with the 15°C trial. The graph was modified from: Guimarães JB, Wanner SP, Machado SC, Lima MR, Cordeiro LM, Pires W, La Guardia RB, Silami-Garcia E, Rodrigues LO, Lima NR. Fatigue is mediated by cholinoceptors within the ventromedial hypothalamus independent of changes in core temperature. Scand J Med Sci Sports 2013; 23:46-56. Copyright © 2013 John Wiley and Sons. Used with permission.

**Figure 5.**
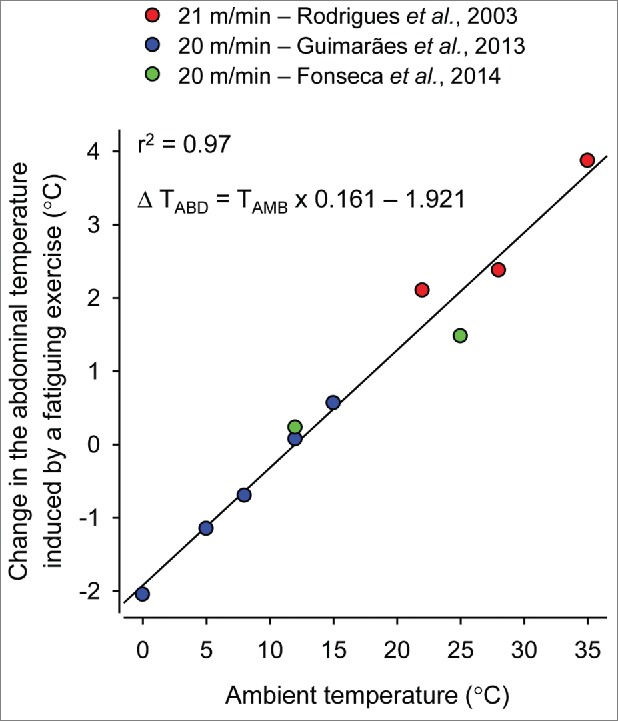
Correlation between the ambient temperature and the change in the abdominal temperature of rats subjected to fatiguing treadmill running. The treadmill speed was set to 20-21 m/min, and the inclination was set to 0-5%. The criterion to determine fatigue was the same in these 3 studies, i.e., the inability to keep the pace with the treadmill, as indicated by exposure to electrical stimulation for 10 consecutive seconds. The data used to plot this graph were taken from experiments reported in the following manuscripts: (i) Rodrigues LO, Oliveira A, Lima NR, Machado-Moreira CA. Heat storage rate and acute fatigue in rats. Braz J Med Biol Res 2003; 36:131-5. Open-access manuscript. (ii) Guimarães JB, Wanner SP, Machado SC, Lima MR, Cordeiro LM, Pires W, La Guardia RB, Silami-Garcia E, Rodrigues LO, Lima NR. Fatigue is mediated by cholinoceptors within the ventromedial hypothalamus independent of changes in core temperature. Scand J Med Sci Sports 2013; 23:46-56. Copyright © 2013 John Wiley and Sons. Used with permission; (iii) Fonseca CG, Pires W, Lima MR, Guimarães JB, Lima NR, Wanner SP. Hypothalamic temperature of rats subjected to treadmill running in a cold environment. PLoS One 2014; 9:e111501. Open-access manuscript.

The existence of a prescriptive zone in exercising rats is unlikely and has never been reported. Even when varying the ambient temperature by a few °C in our previous experiments, we could not observe the existence of a prescriptive zone in the response of the T_CORE_ of rats to treadmill running. As illustrated in **Figure 4**, small alterations in the T_AMB_ (a reduction from 15°C to 8°C) have altered the profile of the T_CORE_ response in rats exercising at the same absolute intensity, with the increase in the T_CORE_ being replaced by a decrease.^103^ Indeed, the data collected from different studies^76,103^^,^^104^ clearly show that the magnitude of the T_CORE_ change induced by fatiguing treadmill running is strongly determined by the T_AMB_ (**Fig. 5**). This relationship was observed at running speeds ranging from 20 to 21 m/min (r^2^ = 0.97; p < 0.001). The fact that the exercise-induced thermoregulatory responses in rats are more sensitive to the T_AMB_ than the responses in humans is explained by differences in their body size. Small animals exhibit a high body surface area-to-mass ratio,^105,106^ which facilitates heat exchange with the environment.

Another relevant aspect regarding the environmental conditions is the wind speed. Some setups are designed with an electrical fan in front of the treadmill belt, whereas others do not have this electrical fan. The airflow generated by the electrical fan is expected to facilitate cutaneous heat loss through convection.^11^ Until a systematic study on the effects of the wind speed on the thermoregulation of running rats is performed, investigators should report whether their exercise setups include an electrical fan and, if so, they should also report the wind speed.

Finally, the effect of different relative humidities on the thermoregulatory responses of exercising rats has never been systemically investigated. The lack of studies on this topic may result from the limited evaporation capacity of rats; Gordon^107^ reported that the evaporation capacity of rats per unit of body mass is half of that of humans. In fact, modern humans possess an extraordinary number of eccrine glands that produce a large amount of watery sweat.^108^ This improved sweating and the resulting higher evaporative capacity are among the anatomo-physiological features that make humans better adapted for long-distance locomotion rather than rapid locomotion and for dissipating heat rather than retaining heat.^109^ Therefore, a higher relative humidity impairs the prolonged physical performance of humans subjected to cycling in hot environments compared with their performance at a lower relative humidity.^110^

Evaporation losses in rodents are dependent on the association between an autonomic (saliva production) and a behavioral thermoeffector (saliva spread onto body surfaces). Saliva evaporation can effectively maintain the T_CORE_ of a rat exposed to heat,^51^ but this process is not effective in an exercising rat because the animal cannot stop running to spread saliva onto its body surface.^37,57^ Nevertheless, despite the limited evaporative capacity of exercising rats, the evaporative water losses (likely water evaporated from the respiratory tract) increase in proportion to the work intensity during 30 min of treadmill running.^53^ This finding warrants future investigations designed to evaluate the impact of different relative humidities on the exercise thermoregulation of running rats.

### The influence of pre-exercise exposure to the treadmill setup

Guaranteeing that rats are not stressed before exercise initiation and determining the resting body temperature values of rats are some of the concerns faced by investigators that study exercise physiology. For example, undisturbed T_CORE_ values of a rat at exercise initiation are essential to obtain recordings showing clear exercise hyperthermia. These resting values are even more interesting if they are recorded while the rats are resting in the treadmill setup. Nevertheless, obtaining such ideal resting values can be challenging and may confound the experimental outcomes.

**Figure 6.**
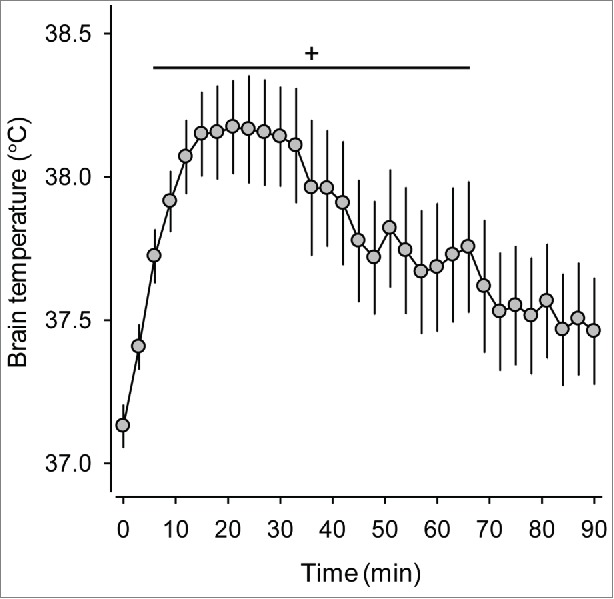
Change in brain temperature induced by manual handling and the insertion of the thermistor for recording the brain cortex temperature (**A**). The rats were kept in their home cages, and the ambient temperature was controlled at 25.2 ± 0.2°C by the use of air conditioning. The data used to plot this graph were taken from the control experiments reported in the following manuscript: Kunstetter AC, Wanner SP, Madeira LG, Wilke CF, Rodrigues LO, Lima NR. Association between the increase in brain temperature and physical performance at different exercise intensities and protocols in a temperate environment. Braz J Med Biol Res 2014; 47:679-88. Open-access manuscript.

**Figure 7.**
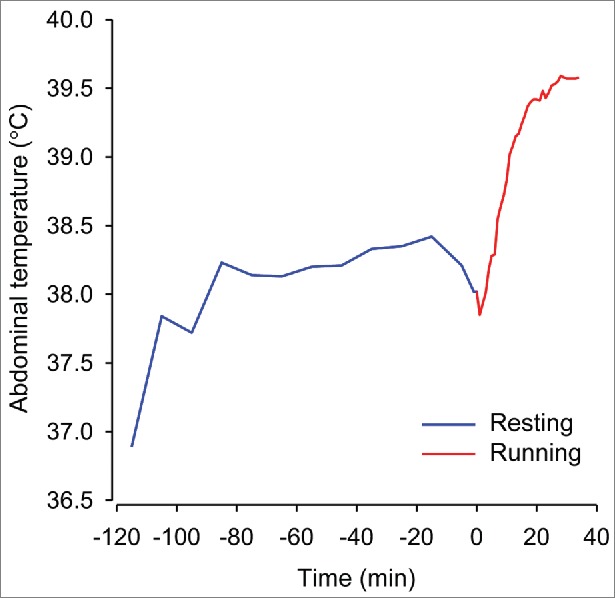
Change in the abdominal temperature of a representative rat that was placed on a treadmill (blue) and 2 h later was subjected to constant-speed treadmill running at 18 m/min (red). A thermocouple remained attached to the rat's tail, and a microinjection needle was placed inside a brain guide cannula throughout the experiment. The ambient temperature was controlled at 24.1 ± 0.4°C by the use of air conditioning. The data used to plot this graph were taken from the experiments reported in the following manuscript: Wanner SP, Leite LH, Guimarães JB, Coimbra CC. Increased brain L-arginine availability facilitates cutaneous heat loss induced by running exercise. Clin Exp Pharmacol Physiol 2015; 42:609-16. © John Wiley and Sons. Reproduced by permission of John Wiley and Sons. Permission to reuse must be obtained from the rightsholder.

After being handled by an experimenter for the insertion of a thermistor that allows T_BRAIN_ measurement, rats display marked but transient hyperthermia: the T_BRAIN_ increases at a rapid rate, peaking at ∼20 min at a value 1.0°C above the initial values, and then decreases slowly toward the initial values (**Fig. 6**). When rats are placed on the treadmill setup before exercise initiation, a consistent and marked increase in the T_CORE_ is also observed (**Fig. 7**). This increase in the T_CORE_ may be a consequence of emotional hyperthermia or may be a conditioned or anticipatory response. The hyperthermia that occurs prior to exercise was first described by Gollnick and Ianuzzo;^90^ when the rats were placed on stationary treadmills, their resting T_COL_ values increased to 39.4°C before the treadmill was turned on. The authors suggested that the pre-exercise hyperthermia represented an anticipatory response that would be elicited by the exercise itself or by the use of electrical stimulation early in the training program. Gollnick and Ianuzzo also suggested that the hyperthermic response would be ablated as the rats become acclimated to the exercise conditions.^90^ This pre-exercise hyperthermia was consistently observed in our experiments even though our rats were familiarized with treadmill running over a period of 5 consecutive days and were minimally exposed to electrical stimulation during the last familiarization sessions.

In experiments in which rats are placed on a treadmill setup prior to exercise initiation, the animals exhibit average pre-exercise T_ABD_ values higher than 37°C^17,111^ and, in some cases, close to 38°C.^36^ In contrast, when the rats are transferred from their home cages directly to the treadmill, their T_ABD_ values are usually lower than 37°C.^75^ The impact of these initial T_CORE_ differences on physical performance, the cutaneous heat loss threshold, and the T_CORE_ at volitional fatigue, among other physiological parameters, has not been investigated.

Interestingly, in some experiments, the increase in the T_CORE_ observed prior to treadmill running is similar to or even higher than the running-induced increase in the T_CORE_ (**Fig. 7**; the maximal T_CORE_ increase during rest and during treadmill exercise was 1.46 and 1.56°C, respectively). This pre-exercise hyperthermia may be physiologically relevant because a state of cognitive fatigue prior to exertion impairs physical performance.^112,113^ In some cases, the T_ABD_ of a rat will not return to the baseline values during a 3-h period of exposure to the treadmill, particularly when a thermocouple is attached to the tail surface of a rat to measure the T_TAIL_. When performing the experiments for a recently published manuscript,^36^ we aborted 35.5% of the experimental trials because the rats exhibited constantly high T_ABD_ values during the treadmill exposure period that preceded running. Taking our experience into account, we recommend that other authors do not expose rats to the treadmill setup in the moments prior to exercise in experiments designed for body temperature measurements even though handling prior to exercise may confound the thermoregulatory response. Future studies should be conducted to further investigate which experimental protocol yields less "noise" when measuring the body temperatures of a running rat.

### Influence of the time of day

The circadian oscillations of the neuroendocrine and autonomic nervous systems directly affect the T_CORE_.^114^ In animals with nocturnal habits, including laboratory rats, T_CORE_ peaks are observed during the early hours of environmental darkness; in contrast, the lowest T_CORE_ values are observed during the light phase of the day.^115^ The circadian rhythm of the T_CORE_ is closely associated with the circadian rhythm of spontaneous locomotor activity^116^ such that metabolic heat production and, consequently, body heat storage increase during the dark phase partially due to an increased metabolic rate in the contracting muscles. In addition, changes in autonomic activity throughout the day modulate the T_CORE_ independently of the locomotor activity rhythm^117^ as the temperature rhythm also reflects the combined effects of the body clock, sleep, feeding, and mental activity.^118^ ^119^ Because the circadian rhythm significantly affects thermoregulation, it would be expected that the exercise-induced changes in body temperature would be dependent on the time of the day at which a laboratory rodent is subjected to physical exertion.

Machado et al.^120^ recently conducted experiments with rats, which were subjected to incremental treadmill running during the beginning of the light or dark phase of the day. As previously observed by other authors,^121^ the pre-exercise T_CORE_ values were lower during the light phase than during the dark phase.^120^ Interestingly, no differences were observed in the T_CORE_ at volitional fatigue between the exercises performed at the 2 phases. This finding suggests that rats show improved heat loss mechanisms and/or running economy in the dark phase. Furthermore, Tanaka et al.^121^ observed higher or unaltered oxygen consumption depending on the exercise intensity but a short latency time for the T_TAIL_ increase during the dark phase; these findings rule out the hypothesis of improved running economy (would be associated with reduced VO_2_) and support the notion that accelerated heat loss may have contributed to the slower increase in the T_CORE_ during exercise at night. An unexpected outcome of the study conducted by Machado et al.^120^ was that physical performance, as measured by the running time to fatigue, was higher during the light phase, which is the phase of the day when rats are less active. An interesting hypothesis to explain the accelerated fatigue in the dark phase is that high pre-exercise T_CORE_ values are associated with reduced prolonged (aerobic) physical performance in both rats and humans,^80,122^ particularly in warm environments.

### Influence of the estrus cycle

Cyclic hormonal fluctuations directly influence body temperature regulation in women. During the luteal phase, which is characterized by increased progesterone levels, the T_CORE_ is approximately 0.3 to 0.5°C higher, and the T_CORE_ threshold for peripheral thermoeffector responses is shifted rightward compared with that observed in the follicular phase.^123,124^ In fact, the measurement of the T_CORE_ is used as a method to verify the occurrence of ovulation. Under exercise conditions, a significantly higher T_REC_ is also observed in the luteal phase, irrespective of the exercise intensity or protocol.^125,126^

Unlike the reproductive cycle of women, the reproductive cycle of female rats, which is known as the estrous cycle, lasts approximately 4-5 d and comprises 4 phases: proestrus, estrus, diestrus and metestrus.^127,128^ These cyclical changes correspond to distinct patterns of hormonal release, which leads to morphological changes that are detectable in vaginal smears.^127^ The most pronounced hormonal changes occur in the proestrus phase, when the luteinizing hormone, follicle-stimulating hormone, prolactin and estradiol concentrations reach their peaks during the estrous cycle.^128^

Female rats exhibit higher T_COL_ values than male rats when the measurements are made 1-2 h before the lights are turned off.^129^ In addition, the T_COL_ of female rats fluctuates throughout the estrous cycle. During the light phase of the day, the T_COL_ is higher at proestrus than at diestrus or metestrus.^129^ A similar trend was observed during the dark phase of the day. Marrone et al.^129^ also reported that gonadectomy significantly lowered the T_COL_, attaining values similar to those observed in diestrus females. Then, 3 doses of progesterone and estradiol benzoate were administered systemically to ovariectomized females. Both hormones transiently increased the T_COL_, and the increase in the T_COL_ was directly related to the progesterone dose but was inversely related to the estradiol benzoate dose.^129^

The findings reported by Marrone et al.^129^ were not reproduced in future investigations. Yanase et al.^92^ observed that the T_REC_ was ∼0.5°C higher in the estrus than in the proestrus stage during the dark phase of the day. In addition, Kent et al.^130^ observed an increase in the nighttime T_CORE_ but a decrease in the daytime T_CORE_ and a higher amplitude of the T_CORE_ rhythm during proestrus compared with the other estrous cycle phases. All of these changes were recorded when the rats had free access to running wheels and were significantly reduced when the running wheels were locked.^130^

When female rats are passively exposed to a warm environment, the threshold T_REC_ for an increase in the T_TAIL_ is also higher in the estrus phase than in the proestrus phase.^92^ In contrast, no differences have been reported between the 2 phases in the threshold T_REC_ and the steady-state T_REC_ at the end of an exercise period. The cutaneous heat loss threshold and T_REC_ at exercise cessation were higher at faster running speeds, regardless of the estrus cycle.^92^ The findings provided by this unique study on exercise thermoregulation throughout the estrous cycle indicate that the T_CORE_ during treadmill running is regulated at a certain level depending on the exercise intensity and is not influenced by the estrus cycle.

## Association between Thermoregulation and Fatigue / Exhaustion

In addition to providing a better understanding of thermoregulation in a condition of increased metabolic rate, the measurement of body temperatures in running rats may also provide information about the mechanisms underlying exercise fatigue / exhaustion. Thermoregulatory responses are among the physiological responses that regulate fatigue / exhaustion and, consequently, prolonged (aerobic) physical performance, especially in the heat.

Fatigue has been traditionally defined as the exercise-induced reduction in maximal voluntary muscle force that may arise not only because of peripheral changes at the muscle level but also because the central nervous system fails to adequately drive motoneurons.^131^ An alternative definition states that "fatigue is a brain-derived emotion that regulates the exercise behavior to ensure the protection of whole body homeostasis" and draws the attention to the fact that fatigue is a protective mechanism rather than a failure of the organism.^132^ Other definitions exist (e.g., the psychobiological model based on motivational intensity theory),^133^ indicating a current lack of a universally accepted definition of fatigue in the scientific literature.

In experiments with rats, fatigue is usually defined as the moment when the animals cannot keep the pace with treadmill^17,134^ and/or expose themselves to electrical stimulation during a predetermined time, which corresponds to 10 s in our experiments.^66,76^^,^^103^ Using the latter criterion, we observed that fatigued rats could right themselves when placed on their backs. In contrast, exhaustion is considered extreme fatigue and usually confirmed by the observation that exhausted rats lose their righting reflex.^135-137^ Different criteria exist for defining exhaustion; for instance, in the study performed by Hasegawa et al.^16^ "exhaustion was considered to have occurred when the rat was unable to keep pace with the treadmill and lay flat on, and stayed on the grid positioned at the back of the treadmill for a period of 30 s despite gently being pushed with sticks or breathed on."

Multiple triggers may explain how thermoregulation influences exercise fatigue / exhaustion.^138^ During exercise in the heat, hyperthermia decreases arousal and increases the rate of perceived exertion,^139^ thereby reducing voluntary activation of the muscles.^140^ Great controversy exists about whether a critical T_CORE_ or a dynamic, anticipatory signal, such as the rate of increase in T_CORE_, is associated with exercise interruption in the heat.^141-143^ Seminal studies with rats performed by Fuller et al.^80^ and Walters et al.^144^ indicated that treadmill running was terminated at a critical temperature, regardless of the preexercise T_CORE_ values. The existence of a critical temperature most likely explains why an elevated T_CORE_ at the exercise initiation is associated with decreased aerobic performance.^80,144^ Whereas, a decreased T_CORE_ is associated with improved aerobic performance;^120^ for example, the fact that the rat T_CORE_ is lower during the light phase gives these animals extra running time to reach the same threshold temperature for fatigue, which does not differ between the light and dark phases of the day. Similarly, improved physical performance is attained by precooling the body before exercise in the heat.^145^

The major problem with the critical end-point theory is that the T_CORE_ values measured at fatigue are not consistent among different studies. Fuller et al. reported that rats fatigued with abdominal and hypothalamic temperature values close to 40.0°C, whereas Walters et al.^144^ reported rectal and hypothalamic temperatures ranging from 42.2 to 42.5°C and from 41.9 to 42.2°C, respectively. This large inter-study variability is supported by the data from Pires et al.,^146^ who observed that rats subjected to an incremental-speed running in the heat exhibited an average T_ABD_ of 40.9°C at fatigue. Therefore, there is not a unique value that limits aerobic performance in rats, as already demonstrated for humans.^143^

Some authors have proposed that exercise is limited by the rate of increase in T_CORE_.^38,104^^,^^141^ However, the claim that hyperthermia is prevented by a feedforward calculation of the rate of body heat storage^141^ is not supported by the available data.^142^ In addition, care should be exercised when analyzing the heat storage or the T_CORE_ data relativized by time (i.e., the rate of heat storage or the rate of T_CORE_ increase), particularly in rats subjected to fatiguing running. When the rate of T_CORE_ increase is analyzed throughout the exercise period, it is faster at higher treadmill speeds than at lower treadmill speeds.^38,104^ Nevertheless, aside from being caused by the thermoregulatory effects induced by distinct running speeds, this difference is also a consequence (and in some cases, an exclusive consequence) of the shorter exercise durations at higher speeds relative to those at slower speeds. Thus, the correlation between the rate of T_CORE_ increase and the running time to fatigue has the disadvantage of including exercise time in both sides of the correlation and, therefore, making this analysis more likely to be significant.

Recently, an alternative hypothesis to explain the decreased aerobic performance in the heat was suggested. Cheuvront et al.^143^ proposed that the cardiovascular adjustments accompanying high skin temperatures, alone or in combination with high core temperatures, provide a primary explanation for the impaired aerobic performance of humans in hot environments. In addition, hypohydration would exacerbate the ergolytic effect promoted by high skin temperature. This hypothesis was not tested in exercising rats, but it may not be applicable to this animal species because rats do not experience significant water losses while running.

Blood flow redistribution represents another possibility through which severe hyperthermia may compromise aerobic performance. During exercise in the heat, blood flow to the gastrointestinal tract is markedly reduced,^147,148^ and this change may compromise the integrity of the intestinal walls, leading to endotoxemia (bacterial translocation from to intestine lumen to the circulation).^149^ This endotoxemia can trigger a cascade of detrimental physiological responses, including a fever-like situation induced by cytokines that accelerates heat storage.^138^ Another relevant physiological consequence of endotoxemia is that contractile proteins may be damaged by the endotoxemia-induced production of reactive oxygen and nitrogen species.^150^

## Comments Regarding Running-induced Thermoregulatory Responses in Mice

The previous sections focused on the exercise-induced changes in the body temperatures of rats. Nevertheless, the use of mice in biological and medical research has been increasing exponentially in the last decades due to the development of important genetic tools in this species, including the development of *knockout* mice. Genetically altered mice play a vital role in understanding gene function and the role of such genes in disease. These mice are particularly important for the creation of animal models of human disease and are therefore vital in the hunt for effective treatments for serious and often life-threatening conditions.

We recently investigated the changes induced by different exercise intensities and protocols in the T_CORE_ of mice.^20^ While designing these experiments, we performed an extensive literature search and could not find a single study that measured the changes induced by a physical exercise bout in the T_CORE_ of mice. As expected, the T_AMB_ greatly influenced the hyperthermia level of running mice, which displayed T_ABD_ values close to 40°C at the end of a treadmill exercise period conducted at high temperatures (34°C). Interestingly, similar hyperthermia was observed in mice subjected to constant- and incremental-speed treadmill running or to constant-speed running at different intensities, irrespective of whether the environment conditions were temperate or hot.^20^

The running-induced increase in the T_CORE_ of mice was not dependent on the exercise intensity (either absolute or relative), suggesting the existence of a regulatory component within the hyperthermic response of exercising mice. Different running speeds induce different metabolic rates^151^ and, consequently, heat production; therefore, heat defense thermoeffectors are likely activated in a manner that allows the T_CORE_ to be regulated at a similar level during the performance of distinct protocols. Thus, physical exercise induces regulated hyperthermia in running mice, and this regulation is characterized by upward shifts in the thresholds for both heat loss and heat production.

The finding that exercising mice present different physiological responses from other species was also obtained in an investigation focusing on their running-induced ventilatory responses,^152^ which may ultimately lead to a distinctive pattern of evaporative heat loss from the respiratory tract. These findings indicate the existence of interspecies differences in the mechanisms underlying exercise hyperthermia and suggest that rat experiments may be more interesting than mouse experiments for studying some aspects of human thermoregulation during exercise.^38^

## Applications of the Outcomes of Rodent Experiments to Human Thermal Physiology

Knowledge of the similarities and differences in morphophysiological features between laboratory rodents and humans is relevant when attempting to apply the outcomes obtained in experiments conducted with rats and mice to human physiology. The objective of the current section is to discuss the application of this knowledge.

Comparison of the baseline parameters show that humans and rats exhibit similar T_CORE_ (∼36-37°C) and skin temperature (∼30-33°C) values under thermoneutral conditions.^107^ However, the resting metabolic rates of rats (expressed as W.kg^-1^ or mLO_2_.kg^-1^.min^-1^) are ∼3- to 5-fold greater than those in humans. The higher resting metabolic rates observed in laboratory rodents is caused by their higher surface area-to-body mass ratio, which is an important parameter that determines the thermal energy exchange between a body and the surrounding environment. A larger surface area-to-body mass ratio is associated with a higher amount of exchanged heat. For example, a 250-g rat has a ratio that is ∼5-fold greater than that of a 80-kg human (0.13 vs. 0.025 m^2^.kg^-1^); thus, a 5-fold higher resting metabolic rate will be required for this rat to maintain T_CORE_ and skin temperature values similar to those of the human in a thermoneutral environment.^107^ A similar rationale can be employed when comparing the thermoregulatory features observed in children and adults.^153^

`Another important inter-species difference is that humans have a greater density of eccrine sweat glands and, consequently, a greater ability to dissipate heat by evaporative means.^109^ These eccrine glands reside close to the skin surface and discharge thin, watery sweat (which vaporizes more readily) through tiny pores directly onto the skin.^108^ Humans exhibit a maximum evaporation rate that is two-fold greater than that exhibited by rats.^107^ In fact, this improved ability to evaporate water from the body surface allows humans to overcome the thermoregulatory challenges of long-distance running. Compared with other mammals, humans have exceptionally better capabilities to run long distances in hot, arid conditions.^109,154^ In an exercising rodent, evaporative heat loss is limited because the animals do not sweat and cannot behaviorally spread saliva onto their body surface while running.^57^ The only means through which rats can rely on evaporation during a period of treadmill running is through the evaporation of water from the respiratory tract.^53^

Although both species have developed distinct strategies to address environmental thermal challenges, the exercise-induced adjustments in heat production and heat dissipation occur in the same direction in both rats and humans.^17,34^^,^^42^ Heat production sharply increases with exercise initiation, whereas the convective cutaneous heat loss decreases until the attainment of a T_CORE_ threshold that triggers skin vasodilation. Thus, the exercise-induced increase in the rate of heat production is always faster than the increase in the rate of heat loss, increasing the T_CORE_ at the beginning of the exercise period in both species.

In addition, rats and humans have a particular skin type, termed glabrous skin (non-hairy) that covers specialized organs for heat exchange with the environment. These organs are located in the most distal parts of the body such as the rat tail^82,155^ and the human hand.^156^ These specialized organs are characterized by a high surface-to-volume ratio, have a dense network of blood vessels, present arteriovenous anastomoses, and thereby function as important radiators and insulators.^156^

Without a doubt, the measurement of skin temperature in the rat tail contributed to advancements in our understanding of the neural mechanisms regulating cutaneous heat loss during physical exercise. However, despite the morphological similarities described above, some differences exist between humans and rats in the innervation of cutaneous vessels. The tail-skin vessels of rats are only innervated by a noradrenergic vasoconstrictor system, and there is no evidence of the existence of an active vasodilator system innervating this cutaneous bed.^46,47^ This pattern of neural control in the rat tail is similar to that observed in the non-hairy (glabrous) skin of humans. In contrast, the hairy skin of humans is innervated by an active, sympathetic vasodilator system, the main neurotransmitter of which remains to be identified.^39,157^ Therefore, the findings yielded from rat studies are more suitable for understanding the brain modulation of heat exchange in the non-hairy skin of humans.

Recent data suggest the existence of relevant inter-species differences in the regulation of regional temperatures, particularly T_BRAIN_. In exercising humans, the jugular venous blood temperature is always higher than the brachial artery and esophageal temperatures during 45 min of cycling at ∼50% of the VO_2MAX_,^158^ thus suggesting that the brain is warmer than other sites of the body core under these conditions. The outcomes yielded from experiments with rats agree with the previous observation; running rats exhibited hypothalamic and thalamic temperatures higher than T_ABD_ throughout the exercise period.^75,76^ However, in response to exercise, the rat T_BRAIN_ increases more rapidly compared with the T_ABD_, irrespective of whether the T_BRAIN_ is measured.^75^^,^^76^ This early difference in the rate of increase in the T_CORE_ indexes likely results from intra-brain heat production. In humans, the venous-arterial temperature difference (i.e., jugular venous temperature – brachial artery temperature) was 0.33°C at rest.^158^ During the first minutes of exercise, the arterial temperature increased at a faster rate than the venous temperature, and the temperature differential was therefore narrowed to ∼0.1°C.^158^ In addition, the VO_2_ in the human brain was not altered throughout incremental exercises.^159,160^ Together, these data indicate that the increase in human T_BRAIN_ during the first minutes of exercise does not result from intra-brain heat production; instead, this increase most likely results from convective heat that is transferred to the brain from the other sites of the body core. Notably, these inter-species comparisons require caution because human data represent changes in the whole brain function, whereas the data collected in rats represent changes in the function of specific brain areas; in this context, important differences in the temperature regulation between distinct brain areas in rats have been reported (**Fig. 2C**).

Finally, the neural pathways controlling autonomic thermoeffectors appear to have been conserved during evolution. Rats and humans have some similarities in the brain areas involved in the modulation of thermoeffector activity. The neural pathways that control tail skin blood flow, brown adipose tissue thermogenesis and shivering in heat- or cold-stressed rats have been elegantly described through a series of studies performed by Nakamura and Morrison.^161,162^ However, the data related to the neural pathways controlling autonomic thermoeffectors in humans remain limited due to methodological and ethical issues. By conducting a functional magnetic resonance imaging study, McAllen et al.^163^ demonstrated that human rostral medullary raphé neurons are selectively activated in response to skin cooling and that the location of these thermoregulatory neurons is homologous to that of the raphé pallidus nucleus in rodents.

## Final Remarks

Collectively, the data presented herein demonstrate the influence of different aspects (environmental conditions, the time of day, and exercise protocol, duration and intensity, among others factors) on the exercise-induced changes in the body temperatures of running rats. These factors should be well controlled to avoid confounders in the results of experiments using rats subjected to treadmill running. The present paper also described some unanswered questions regarding exercise thermoregulation in rats that should be explored in future investigations. Finally, we conclude that studies of running rats can, with certain limitations, help understand some features of exercise thermoregulation in humans.
